# The hydroxyapatite modified 3D printed poly L-lactic acid porous screw in reconstruction of anterior cruciate ligament of rabbit knee joint: a histological and biomechanical study

**DOI:** 10.1186/s12891-023-06245-9

**Published:** 2023-02-27

**Authors:** Yafei Wang, Chengzhen Ren, Fanggang Bi, Pengju Li, Ke Tian

**Affiliations:** 1 Department of Orthopedic Surgery, the First Affiliated Hospital of Zhengzhou University, NO.1 Jianshe East Road, Zhengzhou, China; 2 Department of Orthopedic Surgery, the Honghui Hospital of Xi’an, No. 76 Nanguo road, Nan Xiaomen, Xi’an, 710054 China

**Keywords:** 3D printing, PLLA, Hydroxyapatite, Anterior cruciate ligament, Tendon-bone healing, Biomechanics

## Abstract

**Background:**

3D printing technology has become a research hotspot in the field of scientific research because of its personalized customization, maneuverability and the ability to achieve multiple material fabrications. The focus of this study is to use 3D printing technology to customize personalized poly L-lactic acid (PLLA) porous screws in orthopedic plants and to explore its effect on tendon-bone healing after anterior cruciate ligament (ACL) reconstruction.

**Methods:**

Preparation of PLLA porous screws with good orthogonal pore structure by 3D printer. The hydroxyapatite (HA) was adsorbed on porous screws by electrostatic layer-by-layer self-assembly (ELSA) technology, and PLLA-HA porous screws were prepared. The surface and spatial morphology of the modified screws were observed by scanning electron microscopy (SEM). The porosity of porous screw was measured by liquid displacement method. Thirty New Zealand male white rabbits were divided into two groups according to simple randomization. Autologous tendon was used for right ACL reconstruction, and porous screws were inserted into the femoral tunnel to fix the transplanted tendon. PLLA group was fixed with porous screws, PLLA-HA group was fixed with HA modified porous screws. At 6 weeks and 12 weeks after surgery, 5 animals in each group were sacrificed randomly for histological examination. The remaining 5 animals in each group underwent Micro-CT and biomechanical tests.

**Results:**

The pores of PLLA porous screws prepared by 3D printer were uniformly distributed and connected with each other, which meet the experimental requirements. HA was evenly distributed in the porous screw by ELSA technique. Histology showed that compared with PLLA group, mature bone trabeculae were integrated with grafted tendons in PLLA-HA group. Micro-CT showed that the bone formation index of PLLA-HA group was better than that of PLLA group. The new bone was uniformly distributed in the bone tunnel along the screw channel. Biomechanical experiments showed that the failure load and stiffness of PLLA-HA group were significantly higher than those of PLLA group.

**Conclusions:**

The 3D printed PLLA porous screw modified by HA can not only fix the grafted tendons, but also increase the inductivity of bone, promote bone growth in the bone tunnel and promote bone integration at the tendon-bone interface. The PLLA-HA porous screw is likely to be used in clinic in the future.

## Introduction

Anterior cruciate ligament (ACL) plays an important role in the stability of knee joint and is an important structure to maintain the stability of knee joint [1, 2]. When the tibia is subjected to direct or indirect injury from front to back relative to the femur, it will cause damage to the ACL and lead to knee instability [3]. The incidence of ACL injury in the United States is 1/3000 [4]. Patients with ACL injury are often accompanied by medial collateral ligament injury and meniscus injury, which seriously affect the function of knee joint and restrict the activity of patients [5]. In addition, patients with untreated ACL injuries are at significantly increased risk for secondary cartilage injury and knee osteoarthritis [6, 7]. The most common method of treating ACL injury is ligament reconstruction surgery [8]. Although this technique has achieved good results in clinic, the healing quality of the tendon-bone interface of the reconstructed ligament is only about 40% of the mechanical properties of the normal tendon-bone interface, which makes the ligament reconstruction prone to complications such as loosening and even repeated rupture, so that it is necessary to undergo revision surgery [9].

One of the main reasons for the poor healing of tendon-bone interface is that the interface screws for ligament fixation have low bone induction [10, 11]. The rate of tendon-bone healing is slower than that of screws [12, 13]. After the screws are absorbed, the bone tissue does not grow completely, thus forming cavities, resulting in unstable ligament fixation [14, 15]. In order to achieve an ideal and stable tendon-bone interface, it is necessary not only to synchronize the bone tissue growth rate with screw absorption, but also to have sufficient growth space for new bone to promote bone integration. Therefore, the ideal interface screw should have both the functions of inducing osteogenesis and promoting tendon-bone healing [16].

In recent years, special attention has been paid to screws made of poly L-lactic acid (PLLA), which not only hold the graft ligament firmly in the bone marrow tract, but also reduce the incidence of adverse reactions such as inflammatory reactions and ensure easy biological integration of the graft into the bone tunnel [17]. However, such screws still have limitations, such as the problem of postoperative tunnel enlargement [18, 19], and the need to introduce screws made of materials that are more reliable and closer to the biomechanical properties of metal implants..

Today, 3D printing technology has been successfully applied in various fields [20–22], and its extensive research focus is on the combination of new materials and technologies to enhance product functionality and reduce costs. Related studies have shown that 3D porous screw structure has become one of the important factors in promoting bone formation by providing enough space for the growth of new bone [23, 24].

Therefore, the purpose of this study is to prepare PLLA porous screw using 3D printing technique. Hydroxyapatite (HA) was used to modify the porous screw to enhance bone induction, and to evaluate the effect of HA modified porous screw on tendon-bone healing in ACL reconstruction animal model. We speculate that the porous screw can not only fix the transplanted tendon, but also provide enough space for the bone growth around the transplanted tendon, and promote the osseointegration of the transplanted tendon in the bone tunnel by increasing the inductivity of bone.

## Materials and methods

### Preparation and characterization of PLLA porous screw

The PLLA porous screw was designed with Maipu Medical 3D printer software (Fig. 2a). The screw preparation process was completed at the 3D Printing Center of the First Affiliated Hospital of Zhengzhou University. The screw surface and spatial structure were observed under scanning electron microscope (SEM), and the length and width of statistical pores were measured. A total of 6 specimens were measured. The porosity (P) of porous screws was measured and calculated according to the liquid replacement method, and anhydrous ethanol was used as the moving fluid into the pores of the screws. The calculation is shown below:

$$\textrm{P}=\left(\textrm{V}1-\textrm{V}3\right)/\left(\textrm{V}2-\textrm{V}3\right)\times 100\%,$$

In the above formula, V_1_ is the initial volume of ethanol, V_2_ is the volume after the porous screw is in full contact with ethanol, and V_3_ is the volume of remaining ethanol after the porous screw is removed. A total of 6 specimens were measured.

### HA surface modification of PLLA porous screws

Through electrostatic layer-by-layer self-assembly (ELSA) technology, HA can be adsorbed on PLLA screws to form PLLA-HA porous screws. ELSA takes the electrostatic force between ions as the driving force of adsorption [25, 26], and polycations are commonly used as chitosan (CHI) and polyanions as sodium tripolyphosphate (STPP) [27]. Dissolve CHI in 2% acetic acid to obtain 1% CHI solution. Add HA powder into CHI solution and mix well to get 4% HA-CHI solution. 1% STPP solution was prepared from distilled water. Vacuum immersion of PLLA porous in STPP solution for 20 min to stabilize the negatively charged modified scaffold, followed by washing twice with distilled water under vacuum, and then freeze-drying for 30 min. Soak the porous screw in 1% HA-CHI solution to force the solution into the pore until there are no bubbles in the screw (10 min). Then centrifuge in a centrifuge with a radius of 5 cm (2000 r/min, 5 min). The screws were dried at room temperature for 20 min. The surface and spatial structure of porous screw were observed by SEM.

### Experimental animals and groups

This study has been examined and approved by the Ethics Committee of the first affiliated Hospital of Zhengzhou University (Ethics No.KY-2021-0235). Thirty New Zealand male white rabbits were provided by the Animal Experimental Center of Zhengzhou University (2.0–2.5 kg, 12 weeks). Conduct of experimentation on animals followed the recommendations of the ARRIVE guidelines [28]. The simple random grouping method was used to divide the PLLA group(*n* = 15) and the PLLA-HA group(*n* = 15). Preoperative examination showed that both lower limbs had good mobility, no redness, swelling and deformity, and the anterior drawer test and Lachman test were negative.

### Establishment of a rabbit ACL reconstruction model

**Fig. 1:**
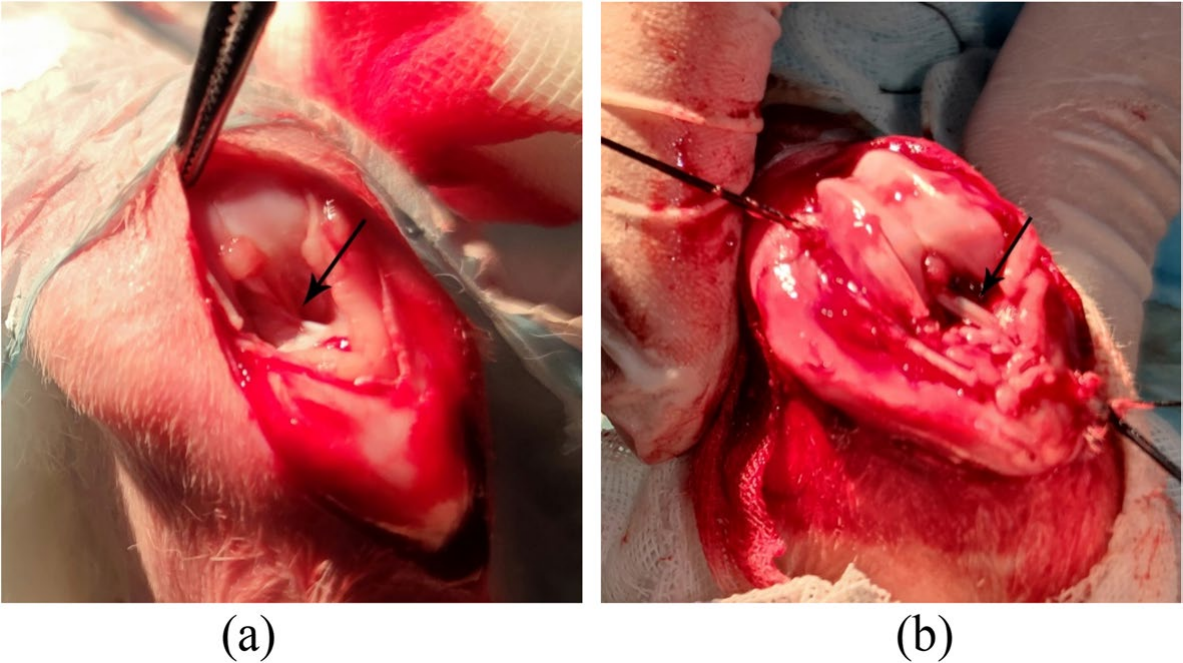
**a** General observation of the rabbit ACL: arrows point to the ACL. **b** General observation of the reconstructed ACL knee joint: arrows point to the implanted graft tendon

The animals were weighed and anesthetized by injecting 3% pentobarbital sodium solution through the ear vein (30 mg/kg). The surgical area was skinned, disinfected and covered with sterile pore towel. The medial incision of the right knee joint was made and each skin layer was incised successively. The semitendinosus tendon was found in the medial tibia condyle of the knee joint, and the tendon was knitted and fixed at both ends with surgical suture and placed in normal saline for use. After exposing the articular cavity, the two ends of ACL were clearly defined, and the ACL was removed at both ends. The grafted tendon was drilled out of the tunnel along the direction of ACL with 2.0 mm Kirschner needle, and the grafted tendon was passed through the bone tunnel. The femoral end was fixed with porous screws, and the tibia end was fixed with sutures. The surgical incision was completely hemostatic and sutured layer by layer. Then the animals were put back in the cage and moved freely. The dressing was changed 3 days after operation. Intramuscular injection of penicillin 300,000 U was given. Euthanasia was performed at 6 and 12 weeks after operation, and the right knee joint was collected for histological, Micro-CT and biomechanical examination (Fig. 1).

### Histological staining

Evaluation of osseointegration at tendon-bone interface by histological staining. Femur and tibia specimens (*n* = 5 in each group) were immersed in 4% paraformaldehyde for 24 hours at 6 and 12 weeks after operation. The specimens were then decalcified in 10% ethylenediamine tetraacetic acid (EDTA) until they were easily cut with a blade. The specimens were embedded in paraffin and sliced horizontally perpendicular to the tunnel axis at the bone-tendon interface. After hydration with ethanol and fixation with Bouin solution, the specimens were stained with Russell-Movat. Then the osseointegration of the graft interface was observed under microscope.

### Micro-CT

The femur-grafted tendon-tibia complex was made from 12 weeks postoperative specimens (*n* = 5 in each group). In order to quantify the mineralized tissue growth, three-dimensional reconstruction of the complex bone tunnel was performed using Micro-CT (μCT-100, SCANCOMedicalAG,Switzerland;30 μm). Each specimen is scanned perpendicular to the long bone axis covering the entrance and exit of the femoral tunnel. The number of trabeculae (Tb.N), bone volume fraction (BV/TV), trabecular thickness (Tb.Th) and trabecular separation (Tb.Sp) were calculated. The measured specimens were placed in a − 80 °C cryogenic refrigerator for biomechanical examination.

### Biomechanical test

The complex was fixed on both ends of the electronic universal material testing machine (Instron 5943，USA, Fig. 7a.) with ligament sutures to ensure that the tibia and femur were in the same coronal plane to prevent the rotation of the transplanted tendon from affecting the mechanical data. The preload was set at 1 N, and the tensile load continuously increased at a displacement rate of 0.5 mm/min and the sampling frequency is 2500 Hz until the tendon grafts are pulled out or broken. The maximum load (failure load) at the time of tendon withdrawal or rupture was recorded. During the test, the specimens were fixed without loosening and kept moist with normal saline all the time. The displacement and failure load were recorded on the load-deformation curve, and the stiffness was calculated by the slope of the curve.

### Statistical analysis

The data were analyzed by SPSS21.0 software, and all quantitative data were expressed as mean ± standard deviation (SD). The differences between groups were tested by independent sample *t-test*. The difference was statistically significant (*P* < 0.05).

## Results

### Electron microscopic observation

**Fig. 2:**
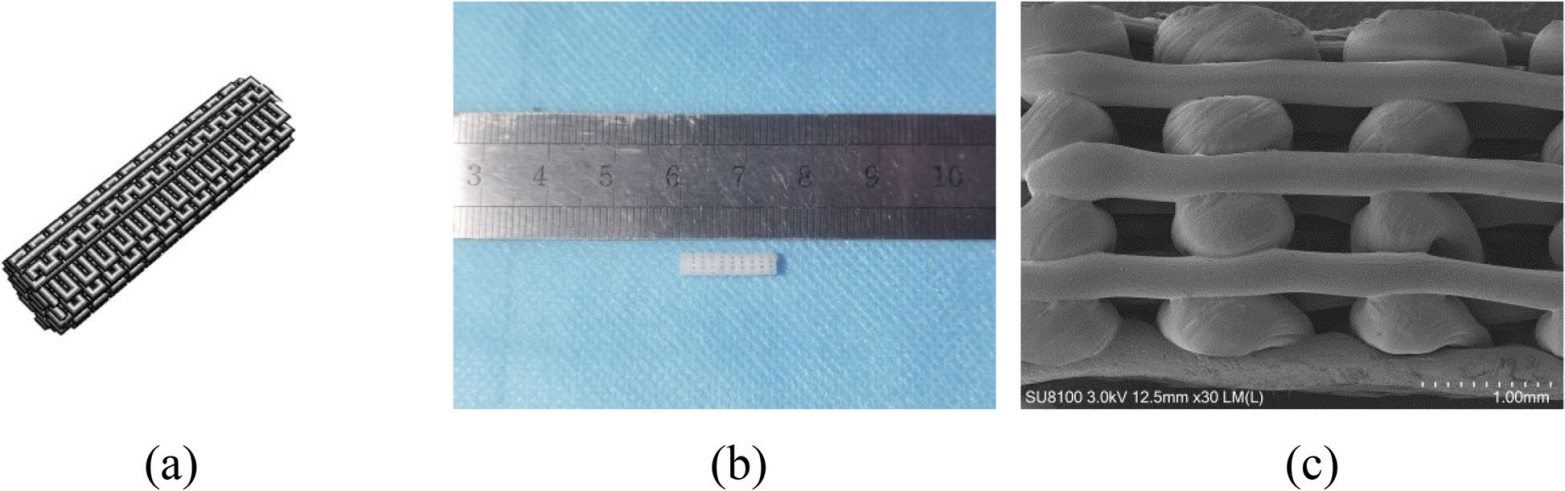
PLLA interface screw support physical properties. **a** the 3D design structure of the PLLA interface screw support. **b** a general view of PLLA screws manufactured by 3D printing. **c** the SEM view of PLLA screw stent shows a uniform and orthogonal stent structure

It was observed that the porous screw had a good orthogonal pore structure under SEM with an average pore length of 410.27 ± 37.44 μm and an average pore width of 332.34 ± 86.82 μm (Fig. 2). The screw porosity determined by liquid replacement method was 44.73 ± 5.58%, which met the experimental requirement.

### HA modified porous screw

**Fig. 3:**
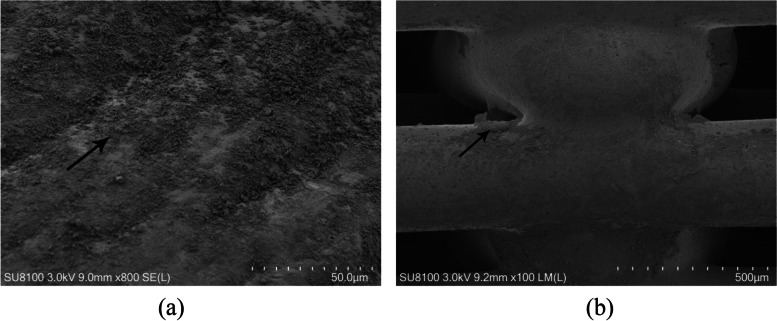
HA was distributed on porous screw. The arrows point to the fact that HA has been distributed on the PLLA surface (**a**) and in the pores (**b**)

It was observed that HA was uniformly distributed on the PLLA porous screw under SEM: not only on the surface of the porous screw, but also in the internal voids of the porous screw (Fig. 3).

### Histological testing

**Fig. 4:**
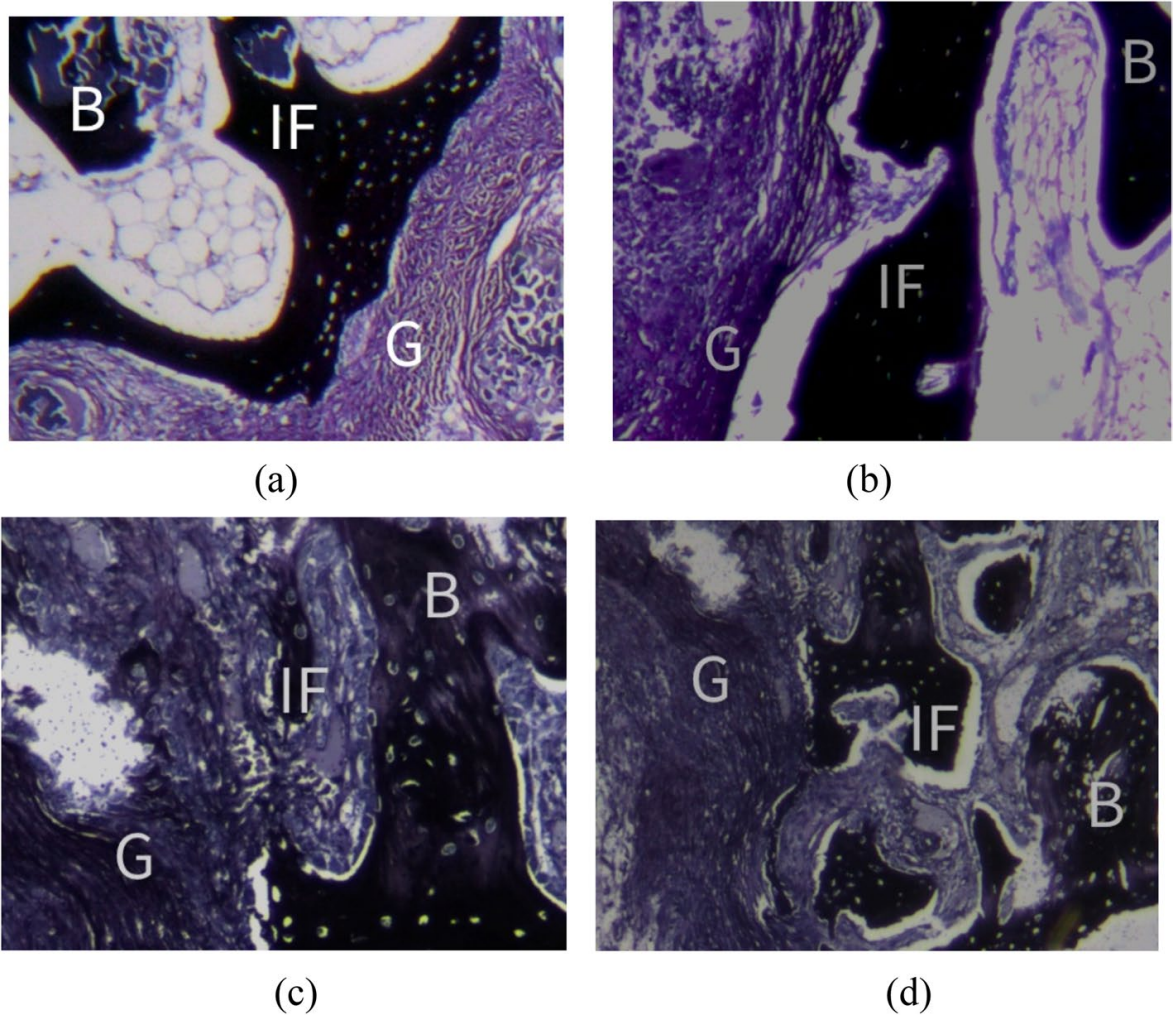
Russell-Movat staining of tendon-bone interface. At 6 weeks after operation, the osseointegration was not obvious in the two groups (**a**,**b**). At 12 weeks after operation, bone trabeculation was observed at the tendon-bone interface in the PLLA group(**c**), while in the PLLA-HA group, there was osseointegration between the bone trabeculae and the transplanted tendons. Scale bar = 100 μm

Histological staining showed that collagen and collagen fibers were produced at the tendon-bone interface 6 weeks after operation, and there was no obvious osseointegration between the two groups, but the chondrocyte layer could be observed in the PLLA-HA group (Fig. 4 a, b). At 12 weeks after operation, more collagen and collagen fibers were produced at the tendon-bone interface. Mature bone trabeculae and cell growth were observed at the tendon-bone interface in the PLLA group, and bonetrabecular integration was observed in the PLLA-HA group (Fig. 4 c, d).

### Micro-CT analysis

**Fig. 5:**
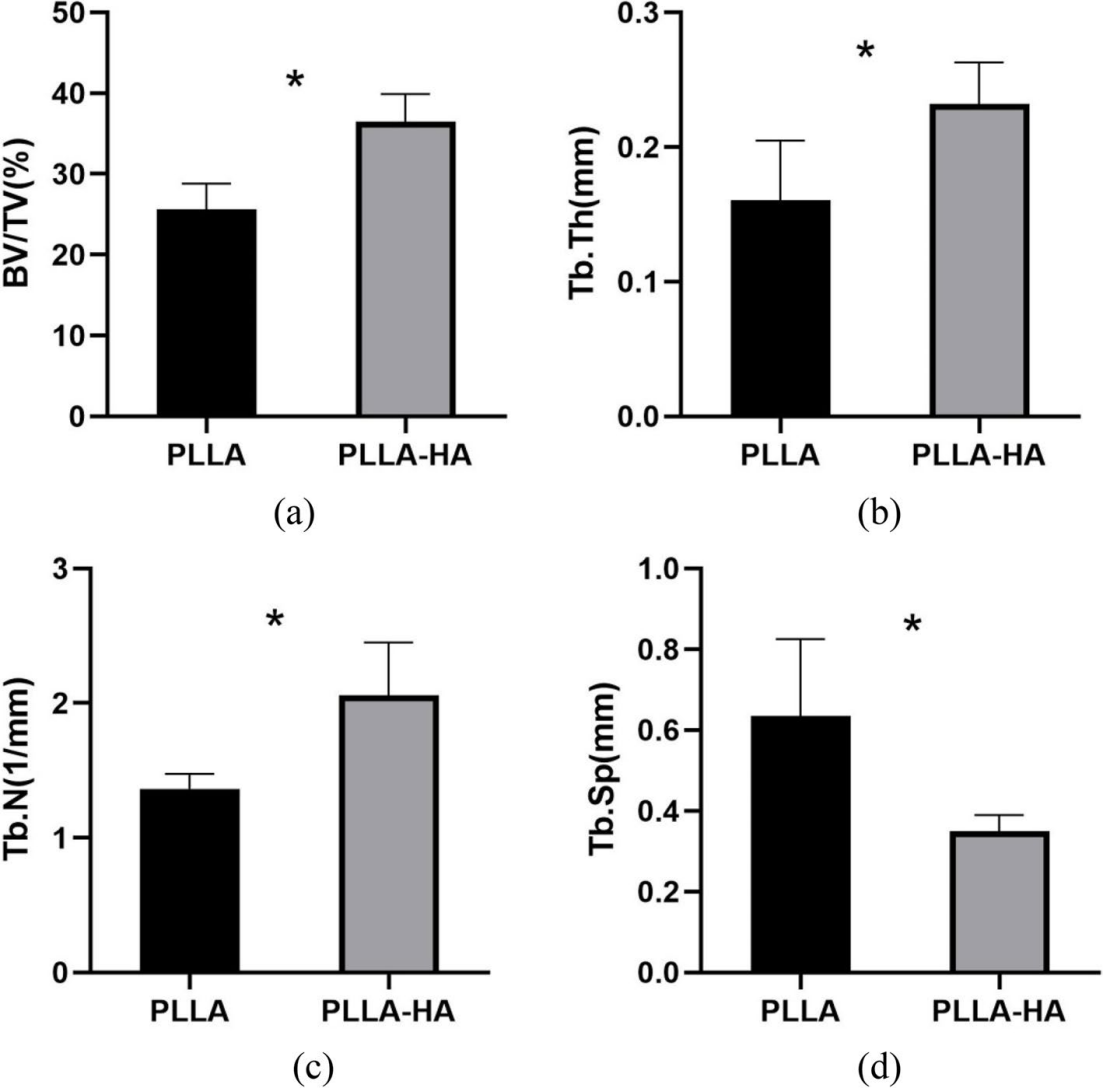
Micro-CT analysis at 12 weeks after surgery. *P < 0.05, indicating a statistically significant difference (**a**: BV/TV, *P* = 0.001; **b**: Tb. N, *P* = 0.005; **c**: Tb. Th, *P* = 0.018; **d**: Tb. Sp, *P* = 0.011)

**Fig. 6:**
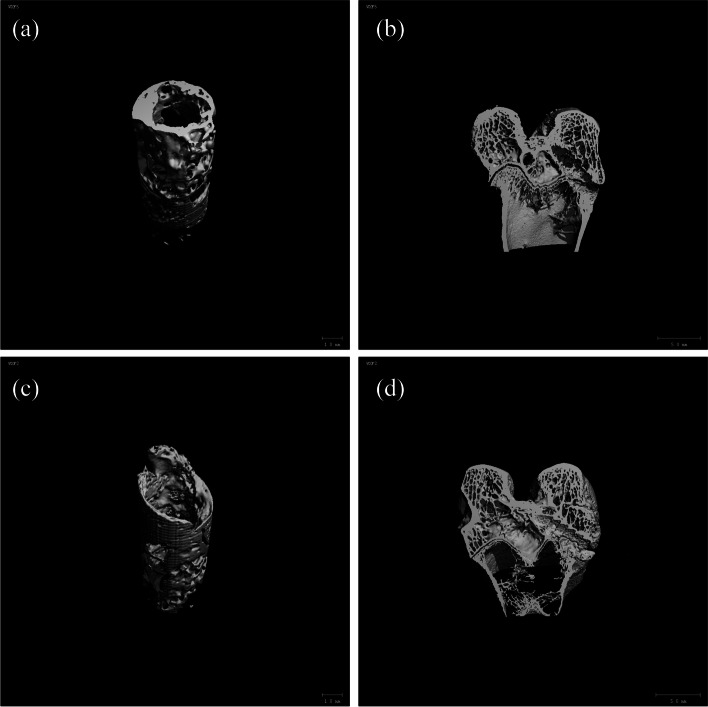
Micro-CT 3D reconstruction of the new bone in the femoral tunnel at 12 weeks postoperatively(**a**, **c**:3D reconstruction image of the femoral tunnel; **b**, **d**: coronal reconstruction)

At 12 weeks after operation, the bone volume fraction (BV/TV) and trabecular thickness (Tb. Th) and trabecular number (Tb.N) in PLLA-HA group were higher than those in PLLA group, while trabecular separation rate (Tb.Sp) was lower than that in PLLA group(*P* < 0.05) (Fig. 5). Compared to the PLLA group (Fig. 6a, b), the new bone in the tunnel of the PLLA-HA group (Fig. 6c, d) exhibited more porous morphology resembling screw structures.

### Biomechanical test and analysis

**Fig. 7:**
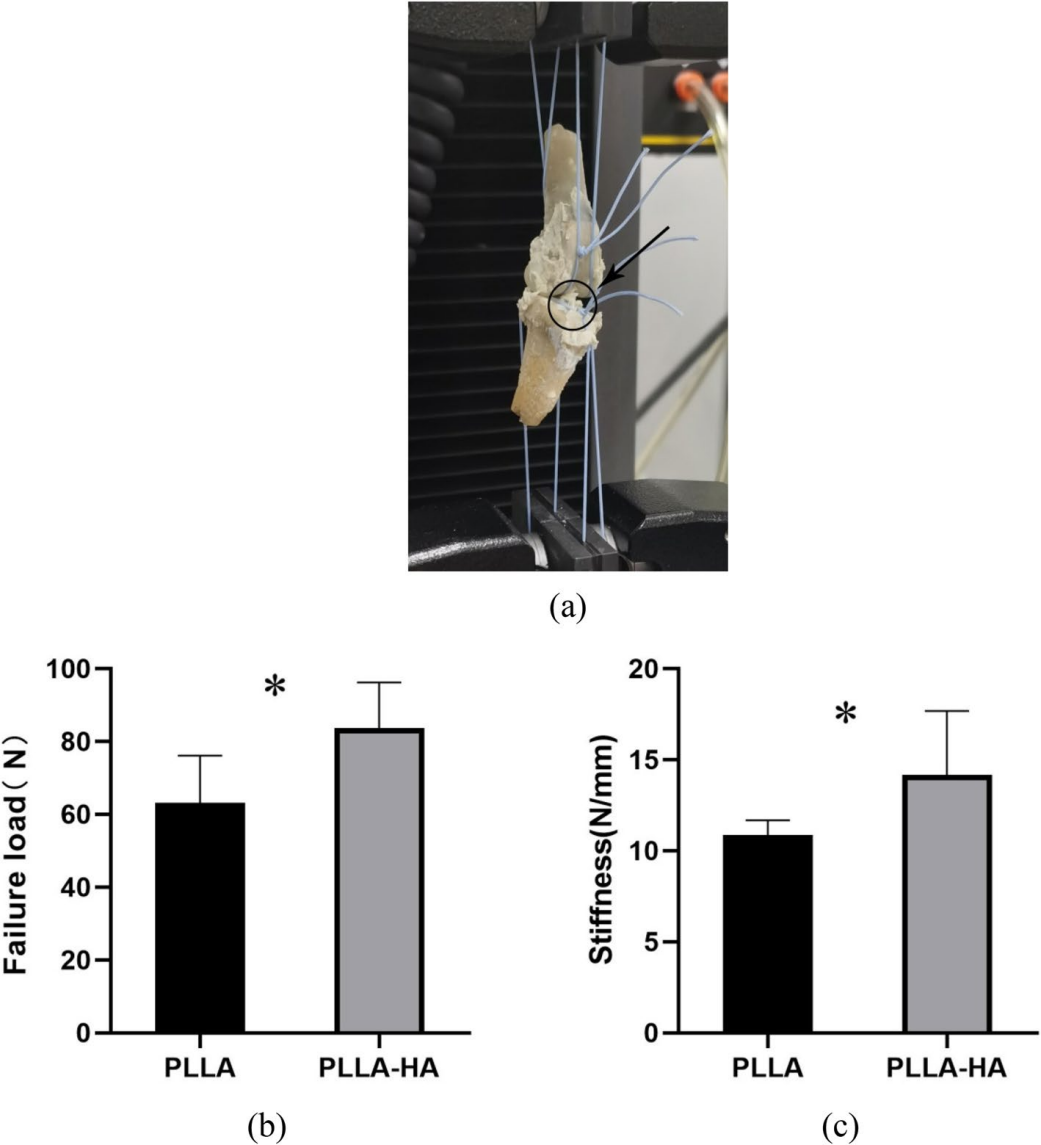
**a** femoral-graft-tibial complex fixation on an Instron machine with ligament suture for biomechanical testing (black arrow points to the graft). **b** The mean failure load at 12 weeks postoperatively was significantly greater in the PLLA-HA group than in the PLLA group. **c** There was also a significant difference in stiffness between the two groups. *P < 0.05 indicates that the difference is statistically significant

At 12 weeks after operation, the transplanted tendons in both groups failed due to rupture in the knee joint cavity or pull out from the bone tunnel. There was a significant difference in failure load between the two groups (Fig. 7b, PLLA 54.70 ± 12.58 N and PLLA-HA 83.74 ± 12.43 N, *P* = 0.006). The stiffness is calculated by recording the displacement and failure load in the load-deformation curve. There was a significant difference in stiffness between the two groups at 12 weeks postoperatively (Fig. 7c, PLLA 10.42 ± 0.71 N/mm and PLLA-HA 14.16 ± 3.53 N/mm, *P* = 0.049).

## Discussion

In this study, the PLLA porous screw was made by 3D printing technology. Surface modification of porous screws by HA can increase the induction of bone and promote the growth of new bone. The porous screws were observed to be regularly aligned with ideal pores by SEM. The successful distribution of newly formed bone tissue in vivo within the PLLA scaffold was confirmed by Micro-CT analysis. The biomechanical test results are also consistent with the radiological results, indicating that the HA modified porous screws play a role in bone induction in the process of tendon-bone healing and promote bone integration. Therefore, the current research results show that HA modified 3D printed porous screws can enhance bone induction and promote bone integration at the tendon-bone interface.

In order to make HA attach to PLLA porous screws, we use ELSA technology. Through electrostatic adsorption, positively charged HA is adsorbed on negatively charged porous screws, thus making HA play the role of bone induction. Guo Peng et al. [29] explored the differentiation of mesenchymal stem cells in the experimental study, which realized sustained gene release through ELSA technology. On the basis of this experiment, Liu An et al. [30] used 3D printing technology to prepare polylactic acid (PLA) scaffold, modified HA on the surface of the stent by ELSA technology, and SEM observed that HA was uniformly adsorbed on the surface of each strut of PLA stent screw. This method is also used in this study to ensure the bone induction of porous screws.

Firm tendon-bone healing is an important basis for ACL reconstruction [9]. Previous studies have shown that osteoinductive agents, such as HA [31, 32], can accelerate osseointegration of tendon grafts and improve tendon-bone healing and mechanical properties [33]. Liu et al. [34] showed that cartilage-like cells could be observed in PLA-HA group at 4 weeks after operation, while the interface of tendon bone in PLA group was fibrous tissue and new bone formation was less. At 12 weeks after operation, the distance between tendon graft and bone marrow tract became narrower, and there were better morphological arrangement of cells and deposition of extracellular matrix, showing a good effect of tendon bone healing. Park et al. [35] found that collagen fiber connections appeared between tendon and bone at 8 weeks, while fibrocartilage appeared around the articular cavity of the bone tunnel at 12 weeks. In this study, at the tendon-bone interface at 6 weeks after operation, collagen fibers and reticular fibers, proliferating chondrocytes and matrix were observed in the PLLA-HA group compared with those in the PLLA group, while at 12 weeks after operation, the continuity of collagen fibers at the transplantation interface increased in the PLLA-HA group, and chondrocytes arranged along the fibers appeared between the fibers, and mature cancellous bone was formed in the core area of the graft, which was more obvious than that in the PLLA group.

Micro-CT identifies subtle changes in bone tunnels and collects broad information about new bone tissue through imaging [36]. In PLLA-HA group, BV/TV,Tb.Th,Tb.N was higher than that in PLLA group, while Tb. Sp was lower than PLLA group. Low Tb. Sp indicates that the trabeculae of new bone are closely arrange. The experimental results indicated that PLLA-HA could promote the formation of new bone in the tunnel, while providing sufficient space for new bone to facilitate the stability of the bone-graft-porous screw structure in the tunnel. This corresponded to the histological presentation. Enlargement of the knee bone tunnel due to bone resorption is a common problem after ACL reconstruction [37]. The newly formed bone promotes direct union between bone and graft and prevents knee instability associated with bone tunnel enlargement. From the current study, it is known that PLLA-HA screws not only provide sufficient space for new bone growth by 3D printing to create interconnected regular porous structures, but also induce osteogenesis and eventually form good tendon bone healing structures. We can predict that new bone formation in the PLLA-HA group may be more effective in terms of long-term function, as it prevents joint instability associated with bone tunnel enlargement.

The pull-out test is objectively detected by biomechanical changes and directly reflects the tendon bone healing process. Anderson et al. [38] showed in a rabbit model of ACL reconstruction that the tendon was still in the early stages of healing and was more likely to be pulled out or loosened by 6 weeks. Tsai et al. [39] studied the application of porous titanium interference screws in rabbit ACL reconstruction, and the failure load was still less than 40 N at 12 weeks after operation. Huang et al. [40] used 3D printed porous titanium screw to fix rabbit tendon grafts, and the failure load reached 56.6 ± 4.7 N at 24 weeks after operation, which was much less than the failure load in this study. The increase of failure load means more bone growth and tendon-bone integration, indicating that PLLA porous screws are much better than titanium screws. The reason for the analysis may be related to the following two points: on the one hand, the screw design has an interconnected porous structure, which may allow neovascularization and bone growth and accelerate tendon-bone integration, on the other hand, HA gives full play to bone induction, promotes more fibroblasts, reticular fiber and bone trabecular formation, and improves biomechanical properties. This corresponds to histological and imaging findings.

In this study, we attached HA to PLLA porous screws using ELSA technique and evaluated the changes in bone volume after grafting using Micro-CT and histology as well as mechanics, but there were shortcomings. Other studies have used more advanced imaging techniques, such as MRI scans, to provide data on the impact of bone tunnel structure after ACL reconstruction, and these methods can assess whether the tunnel widens after grafting [34]. Moreover, the constructed rabbit model and the designed 3D screw scaffold are not representative of the human physiological condition. In addition, previous studies have used cell biology approaches and bioactive materials to promote tendon-bone healing, such as: bone morphogenetic protein (BMP) [41–43], gene therapy [44–46], bone marrow mesenchymal stem cells (BMSCs) [47–49], platelet-rich plasma (PRP) [50–52], and hyaluronic acid [53], all of which are able to influence tendon-bone healing after ACL reconstruction of the knee. In future studies, biological influences will be combined with 3D printing technology in order to better investigate the mechanisms of tendon-bone healing processes.

## Conclusion

HA modified 3D printed porous screw can not only fix the transplanted tendon, but also effectively increase the new bone mass in the bone tunnel and promote the bone integration at the tendon-bone interface by promoting the bone growth in the bone tunnel. HA modified PLLA porous screw is likely to be used in clinic in the future.

## Data Availability

All the data of the manuscript are presented in the paper or additional supporting files.

## Abbreviations

ACL: Anterior cruciate ligament
PLLA: poly L-lactic acid
HA: Hydroxyapatite
ELSA: electrostatic layer by layer self assembly
CHI: chitosan
STPP: sodium tripolyphosphate
SEM: scanning electron microscope
EDTA: Ethylenediaminetetraacetic acid
BV/TV: Bone volume fraction
Tb.N: Trabecular number
Tb.Sp: Trabecular separation
Tb.Th: Trabecular thickness;polylactic acid: PLA
